# Anti-allergic activities of Umbelliferone against histamine- and Picryl chloride-induced ear edema by targeting Nrf2/iNOS signaling in mice

**DOI:** 10.1186/s12906-021-03384-1

**Published:** 2021-08-27

**Authors:** Younas, Ashrafullah Khan, Omer Shehzad, Eun Kyoung Seo, Alev Onder, Salman Khan

**Affiliations:** 1 Pharmacological Sciences Research Lab, Department of Pharmacy, Faculty of Biological Sciences, Quaid-i-Azam University Islamabad, Islamabad, Pakistan; 2 Department of Pharmacy, Abdul Wali Khan University, Mardan, Pakistan; 3 College of Pharmacy, Ewha Womans University, 52, Ewhayeodae-gil, Seodaemun-gu, Seoul, 03760 South Korea; 4 Department of Pharmacognosy, Faculty of Pharmacy, Ankara University, Ankara, Turkey

**Keywords:** Umbelliferone, Picryl chloride, Oxidative stress, Antioxidant, Inflammation

## Abstract

**Background:**

The current study was aimed to investigate the anti-allergic activities of the Umbelliferone (UMB) against the acute Histamine and chronic Picryl chloride (PiCl)-induced allergy in mice. UMB is a coumarin derivative (isolated from *Angelica decursiva*) found in various parts of the plants such as flowers, roots and, stems isolated from the plants of Umbelliferae family.

**Methods:**

The UMB (1, 10, 50 mg/kg) was administered intraperitoneally (i.p) half an h before or 2 h after the induction of allergic ear edema. The acute ear edema was induced by histamine (intradermally, i.d), while the chronic ear edema was induced by painting the PiCl (sensitized with the toluene) on the ear. The antioxidants and oxidative stress markers were assessed. The histological changes were assessed using Hematoxylin and eosin (H and E) and giemsa staining. The immunohistochemistry studies were performed to assess the expression of the nuclear factor erythroid 2-related factor 2 (Nrf2) and inducible nitric oxide synthase (iNOS). The data was analyzed using one-way ANOVA tests followed by Tukey’s test with *p* < 0.05 was chosen as criteria for statistical significance.

**Results:**

UMB treatment markedly reduced the allergic ear edema and ear weight compared to the negative control. Furthermore, the UMB attenuated the oxidative stress markers, while induced the antioxidants enzymes. Similarly, the UMB treatment significantly attenuated the serum immunoglobulin E (IgE) level. The UMB treatment markedly improved the histological parameters using H and E staining and Giemsa staining. The UMB administration induced the Nrf2 expression, while attenuated the iNOS expression. Furthermore, the computational analysis was performed to assess the interaction of the UMB with the various protein targets and to determine the mechanism of interaction with the target proteins.

**Conclusion:**

In conclusion, the UMB treatment significantly alleviated the allergic symptoms, attenuating the oxidative stress, improved the histological features using in vivo and computational approaches.

## Backgroud

Skin is the first and foremost principal barrier to external injury or infectious agent [1]. Usually, the skin offer protection against infectious agents and other harmful agents by synthesizing various chemicals without disturbing the normal tissue [1, 2]. However, disturbance of the immune system may worsen the pre-existing skin inflammatory disease like psoriasis and atopic dermatitis [1, 2]. Atopic dermatitis is an allergic inflammatory condition affecting mainly the keratinocytes of the skin characterized by pruritus, chronic eczematous plaque and relapse upon exposure to an antigen [2]. The inflamed skin shows different histological parameters like scaling, dryness, skin eruption and papules involving eosinophils, mast cells and other inflammatory cells. The disparity of T helper 1 (Th1) and T helper 2 (Th2) cells have been reported in atopic dermatitis with the dominancy of the T helper 2 responses, which leads to the increase production of IgE antibodies [2, 3].

The pro-inflammatory mediators (IL-1β, IL-6 and TNF-α) and oxidative stress plays a critical role in the development of allergic inflammatory disorders. During allergic conditions the NO production is significantly enhanced and its production is regulated by the iNOS genes [2, 3]. The NO production is directly associated with the severity of the allergic symptoms. Increase in the production of NO and oxygen free radicals which may further enhance the vasodilation and cytotoxicity leading to allergic ear edema [2]. The Nrf2 is an important signaling pathway involved in the regulation of oxidative stress and induction of antioxidants enzymes [4]. The Nrf2 remains dormant within the cytosol under the inhibitory influence of Kelch-like ECH-associated protein 1 (Keap1). During basal conditions small only amount of Nrf2 is released and undergoes Proteasomal degradations. During inflammatory conditions, the Nrf2 becomes free from the inhibitory influence of the Keap1 and translocate to the nuclei to interact with the concerned genes [4]. The Nrf2 interacts with the antioxidants response element (ARE) and induces the antioxidants enzymes such as Heame oxygenase-1 (HO-1), Superoxide dismutase (SOD), Catalase, reduced glutathione (GSH), and Glutathione S-transferase (GST) [4]. The antioxidants thus produced neutralizes the oxidative stress markers such as malondialdehyde (MDA) and glutathione peroxidase (GPx) [3]. Similarly, the Nrf2 signaling also inhibit the production of the pro-inflammatory cytokines and attenuate the inflammation [3, 5].

The therapeutic approaches available for treating allergic dermatitis are associated with various side effects like ulcer, sedation and palpitation [5, 6]. The natural products are cheap and important sources of the new drug development and many drugs from natural sources are currently in clinical practice [3, 6, 7]. The Umbelliferone is 7-hydroxycoumarin isolated from the *A. decursiva* and found in various parts of the plants such as leaf, roots and fruits of Umbelliferae family. Chemically it is benzopyrone in nature and reported for a wide range of biological activities [8]. The Umbelliferone have been reported for the anti-inflammatory and antioxidants activity in a chronic animal model of alcohol fed rats and streptozotocin-induced diabetes [4, 9]. Similarly, the anti-cancer and anti-apoptotic activity of UMB have also been documented in the various model of carcinogenesis i.e. colon, hepatic and renal cancer [10–12]. Furthermore, the Umbelliferone have also been implicated in the amelioration of the LPS-induced bone loss and osteoclastogenesis via multiple signaling mechanisms [13, 14]. The ischemic-reperfusion myocardial injury was prevented by the Umbelliferone treatment by suppressing the NLPR3 inflammasome and inducing PPAR-γ. The Umbelliferone have also been reported for anti-arthritic and hepatoprotective activity pre-clinically [15, 16]. Based on the previous reports, it was anticipated that Umbelliferone treatment will ameliorate the allergic ear edema induced by the PiCl (sensitized with Toluene) and Histamine. Furthermore, in the present study, it was aimed to explore the underlying mechanism of Umbelliferone against the PiCl and Histamine-induced allergy.

## Methods

### Chemicals and reagents

The chemicals and reagents used in the current study include UMB, PiCl, histamine, ketamine, dimethylsulphoxide (DMSO), Toluene, dexamethasone, acetone, ethanol, 5,5′-dithiobis-(2-nitrobenzoic acid (DTNB), 1-chloro-2,4-dinitrobenzene (cDNB), phenylenediamine and Tris-HCl were obtained from the Sigma Aldrich (Sigma Aldrich, USA). The avidin-biotin complex (ABC), proteinase-K, 3,3-diaminobenzidine (DAB), xylene, and mounting media were purchased from the Santa Cruz (Santa Cruz, Inc). All the chemical and reagents used in the present study were of analytical grade. The PiCl solution was prepared in acetone and ethanol at 3:1 ratio, respectively.

### Plant materials

The Umbelliferone is 7-hydroxycoumarin in nature and mainly found in the plants of Umbelliferae family [3, 8]. The plant of *A. decursiva* was collected and identified at Korean Research Institute of Bioscience and Biotechnology, Republic of Korea (Lot#012–043, 2006) and the voucher specimen (2006 CHOI-Angelica02) was deposited. The Umbelliferone was isolated and characterized at Pukyong National University (Department of Food and Life Sciences), Busan, Republic of South Korea and [8, 13]. The UMB and dexamethasone was dissolved in the normal saline using 2% DMSO and administered by the intraperitoneal route (i.p) as reported previously [16–18].

### Animals

Male and female albino BALB/c mice (5–7 weeks old) weighing 28–36 g were procured from the national institute of health (NIH) Islamabad. The animals were acclimatized to experimental environment for 7 days before the study and the activities were performed in the pathogen free area i.e. lab of pharmacology, department of pharmacy. The animals were provided 12 h of light/dark cycle, room temperature of 23 ± 0.5 and relative humidity of 55%. The animals were provided free access to water and food. All the procedure were performed according to guidelines of the animal ethical committee of Quaid-i-Azam University, Islamabad (#BEC-FBS-QAU2019–181). During the whole study, great care was initiated to avoid any harm to the experimental animal and number of animals were kept as minimum as possible. During the current study, fresh animals were procured and used once [19].

### Grouping and study protocols

Animals were randomly assigned into six groups randomly (*n* = 5) such as normal control (normal saline 0.9% with 2% DMSO), positive control (dexamethasone 10 mg/kg), negative control (histamine 10 mg/kg in case of acute study, while PiCl (sensitized with Toluene in chronic study)) and UMB (in three different doses i.e. 1, 10 and 50 mg/kg). PiCl was painted topically on right ear of each mouse through micropipette, while UMB was administered through i.p route. Normal control group was treated by painting normal saline on the right ear [19, 20]. For sampling, the animals were anesthetized using combination of Xylazine and Ketamineinjection (16 mg + 60 mg, i.p) to avoid distress and discomfort to the animals during sampling. When the sampling was performed the animals were euthanized using CO_2_ chamber and the animals death was confirmed by assessing the heart rate, eye reflexes and respiration [21]. The institutional ethical committee regulated the overall process of euthanasia.

### Induction of ear edema by histamine (acute study)

The acute ear edema was induced by injecting the histamine intradermal (i.d) in both male and female mice. The edema was induced in the right ear lobe of each group by administering 5 μl of histamine using a special 29 gauge hypodermic needle, while the left ear received 5 μl of saline topically. After 15 min the positive control group was treated with dexamethasone (10 mg/kg, i.p), while the treatment group received UMB (1, 10 and 50 mg/kg, i.p). The ear edema was determined with thickness gauge meter after 2 h of histamine administration as described [19, 20].

### PiCl (sensitization of allergy with toluene) multiple administration induced allergy

**Fig. 1:**
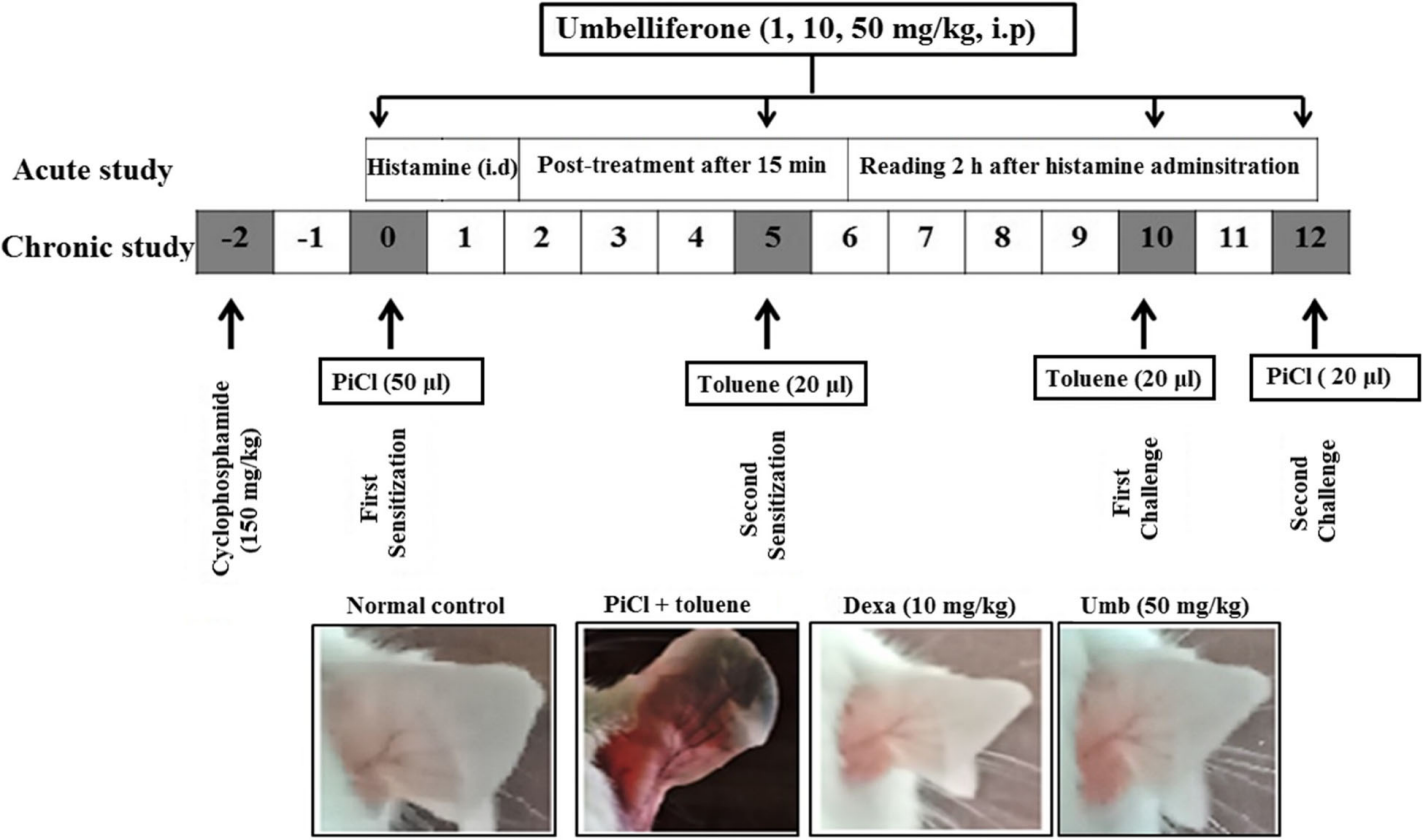
The schematic representation of the current study

Cyclophosphamide was injected subcutaneously (s.c) at a dose of (150 mg/kg) body weight for inducing blood eosinophilia in both male and female mice as described previously [20]. After 2 days i.e. on day 0 the right ear lobe of each group except normal control, were painted by 50 μl of 7% (w/v) PiCl solution (3:1 in acetone:ethanol solution) for sensitization. Following initial treatment, the same groups were treated twice with 20 μl of Toluene solution at the same site on day 5, 10 and 12. Ear edema was measured by dial thickness meter either half an h before or 2 h after inducing allergic disease [22]**.** On day 12, the both male and female mice were anaesthetized with the combination of xylazine and ketamine (16 and 60 mg/kg respectively, i.p) and the whole ear was collected [20, 23]. From the whole ear, 5–6 mm diameter was obtained via metallic punch and weighed on an electronic weight balance for determination of ear mass (chronic study only) [20, 22]. The Fig. 1 shows the schematic representation of the study.

### Assessment of ear edema

Ear edema in all the treated groups (both male and female) were measured with Oditest dial gauge caliper either half an h before or 2 h after induction of allergic edema by histamine (acute study) or PiCl-induced sensitized with Toluene (chronic study) as reported previously [20].

### Determination of skin severity scoring

The severity of allergic ear edema was evaluated in both male and female on every 3rd day by scoring the symptoms following induction of allergic edema [24]. Every symptom was graded (0 = no symptom; 1 = mild; 2 = moderate; 3 = severe) based on evaluation of skin dryness, eruption and wound appearance on right ear of each group reported previously [24].

### Determination of mice weight

The changes in the body weights of all the recruited groups were determined during the whole study after every 3 days for 12 days period. For the determination of the mice weights, the mice were placed on a digital weighing balance [25, 26].

### Weight lifting and light chain assay

To assess the effect of UMB treatment on the muscle coordination and strength following PiCl-induced allergy, weight lifting and inverted mesh screen assay was performed in both male and female as reported previously [26]. The animals were allowed to lift the weight in decreasing order for 3 s and quantify the maximum weight lifted by the mice as reported [26]. Similarly, for the assessment of inverted mesh assay animals were placed on the mesh and inverted to assess the strength of animal holding with the mesh screen as reported [26].

### Determination of biochemical and hematological parameters

The effect of the UMB treatment on vital organs such as liver and kidney were evaluated by observing the biochemical parameters like alanine aminotransferase (ALT), aspartate aminotransferase (AST), bilirubin and creatinine level in plasma following PiCl-induced allergic edema [27, 28]. Similarly, the effect of UMB treatment on the hematology (total leukocytes count and differential leukocytes) was assessed following PiCl-induced allergic edema [27]. Furthermore, the changes in the level of serum IgE in both acute and chronic study was measured by an ELISA kit according to manufacturer instructions (Abcam, USA) [26, 28, 29].

### Determination of antioxidants

The influence of UMB on various antioxidants like reduced Glutathione (GSH), Glutathione S-transferase (GST), Catalase and Superoxide dismutase (SOD) were noted by the method as described previously [30]. The reduced Glutathione (GSH) was performed by taking 100 μl of supernatant obtained from previously homogenized tissue in a 96 well tube [30]. To make the final volume up to 3 ml, 0.5 ml DTNB was added to 2.4 ml of phosphate buffer solution with 0.1 ml of tissue supernatant [31]. The absorbance was recorded at a wavelength of 412 nm. Similarly, the GSH assay was performed by mixing of o.1 ml of supernatant, cDNB and 0.1 M phosphate buffer (pH = 6.5) to make the final volume up to 3 ml. The absorbance was recorded at a wavelength of 314 nm as described previously [31]. This Catalase assay was performed by taking 3 ml of H_2_O_2_ phosphate buffer in a cuvette with 40 μl of tissue enzyme extract and the absorbance was recorded at 240 nm [30, 31]. Similarly, SOD assay was performed by the mixing of 10 μl of sample with the 50 mM Tris-EDTA buffer (pH = 8.5) and 24 mM Pyrogallol to make the final volume up to 0.2 ml. Enzyme activity was measured at 420 nm and expressed as unit/mg protein [30–32].

### Determination of LPO, MPO and EPO

Lipid peroxidation product i.e. MDA was determined in all the study groups following histamine and PiCl-induced ear edema as reported [31]. The reaction mixture consisting of TCA (trichloroacetic acid), TBA (thiobarbituric acid), ferric chloride (FeCl3) and study samples [31]. The reaction mixture was boiled at 100 °C and analyzed using microplate reader at 420 nm. Furthermore, the MPO (Myeloperoxidase) assay was determined in ear homogenate as described somewhere else [33]. The reaction mixture was obtained by the addition of o-dianisidine (16.7 mg) in 90 ml distilled water to which 10 ml of 500 mg CTAB (previously dissolved in PBS) was added. Thereafter, 50 μl of H_2_O_2_ was added to the final reaction mixture along with 7 μl of tissue sample. The Activity was measured by the changes in optic density at 450 nm using microplate reader. The EPO serves as a marker of eosinophilic infiltration and increase concentration have been reported during allergic conditions [33]. The effect of UMB treatment on the EPO activity following chronic PiCl-induced ear edema was evaluated as reported previously [33, 34].

### Determination of NO level

This effect of the UMB treatment was assessed on the NO production following histamine and PiCl-induced ear edema as reported [35]. The NO production was determined in both plasma and tissue homogenate using the Griess reagent method as reported [35]. The NO production was recorded as 560 nm using a microplate reader [35].

### Histological analysis

The H and E staining was performed to assess the histological changes in ear tissue following PiCl-induced ear edema as reported previously [36, 37]. Increase or decrease in the number of various inflammatory cells, changes in cartilaginous diameter and edema (epidermal and dermal) was evaluated in all the treated groups [36]. Similarly, the Giemsa staining was performed to assess the changes in eosinophilic infiltration in ear tissue of all the treated groups. The Giemsa stained images were visualized with the help of light microscope (400X magnification) [36].

### Immunohistochemistry of Nrf2 and iNOS

The immunohistochemistry assay was performed to assess the Nrf2 and iNOS expression following histamine and PiCl-induced ear edema in all the treated groups [38]. The paraffin embedded tissue were deparraffinized in the xylene, ethanol and then washed with the distilled water [37]. Following deparraffinization, the tissues were treated with the proteinase-k, normal goat serum and primary antibodies (kept overnight to allow the interaction of the antibodies with the tissues). Next day, the primary antibodies were washed with PBS and tissue were treated with secondary antibodies [38]. The avidin-biotin complex was applied 2 h after incubation with secondary antibodies and thereafter, the slides were treated with DAB solution as reported previously [38]. The images were then analyzed with microscopes, while the Nrf2 and iNOS expression level was quantified using Image_J software [37].

### Docking analysis

The molecular docking analysis was performed to assess the interaction of the UMB with the IL-6, IL-1β, TNF-α, IgE, iNOS and Nrf2 using autodock vina [39]. The IL-6, IL-1β, TNF-α, IgE, iNOS and Nrf2 proteins were downloaded from the Protein data bank (PDB) [39]. The ligand was prepared using Chemdraw_14.0 and saved as PDB format. The results were visualized using discovery studio visulaizer_16 and protein ligand interaction profiler online server [39, 40].

### Validation of docking

The docking process of the UMB with the studied protein was validated and the root mean squared deviation (RMSD) was determined using LigRMSD software. The co-crystalized ligand was extracted and redocked with same protein and the RMSD value was calculated [39, 40].

### Pharmacokinetic analysis of Umbelliferone

The pharmacokinetics analysis of the Umbelliferone was performed to determine the pharmacokinetic behavior of the UMB [39, 40]. The Swiss target prediction software (http://www.swissadme.ch/index.php) was used to assess the ADME (absorption, distribution, metabolism and excretion), bioavailability, physicochemical characteristics, lipophilicity and drug likeness behavior was analyzed as reported previously [39, 40].

### Statistical analysis

Result obtained was expressed as mean ± standard deviation. One way ANOVA test was performed followed by Post-hock test. *P* value < 0.5 was considered as criteria of statistical significance. ###*P* < 0.001 indicates significant difference of negative control group. The Sigmaplot version_12 was used to plot the results of the study.

## Results

### Ear edema

**Fig. 2:**
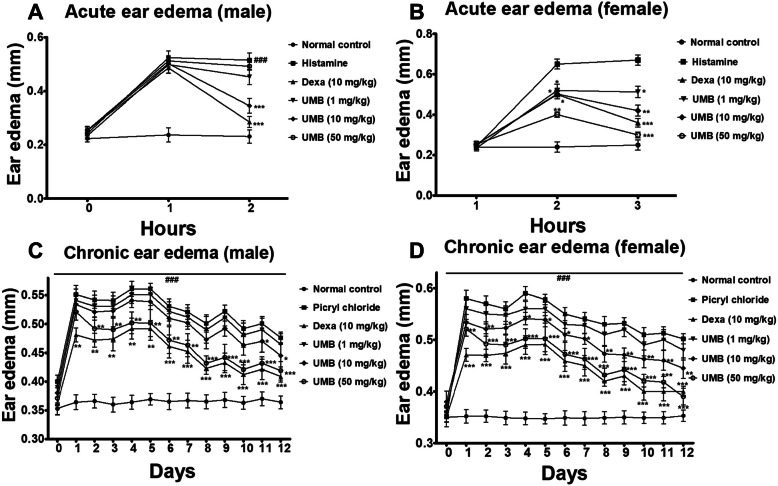
The effect of UMB (1, 10 and 50 mg/kg) treatment on the allergic ear edema (both male and female mice) induced by (**a**, **b**) acute histamine and (**c**, **d**) chronic PiCl (sensitized with Toluene). The UMB dose dependently inhibited the ear edema in both acute and chronic study compared to the negative control. Similarly, the positive control treated with the dexamethasone (10 mg/kg) also significantly attenuated the ear edema. The data were represented as mean ± standard deviation (*n* = 5). (*) *p*<0.05, (**) *p*<0.01, (***) *p*<0.001 indicates significance, while ### shows comparison with the negative control

The ear edema was determined in all the treated groups following histamine and PiCl administration. The negative control (histamine and PiCl treated groups) showed significant increase in the ear edema in both male and female mice. However, the UMB treatment dose dependently attenuated (*P* < 0.05) the ear edema in both acute histamine and chronic PiCl-induced models. Similarly, the dexamethasone treatment also reduced the ear edema and hence, inflammation compared to the negative control as shown in Fig. 2.

### Skin severity scoring

**Fig. 3:**
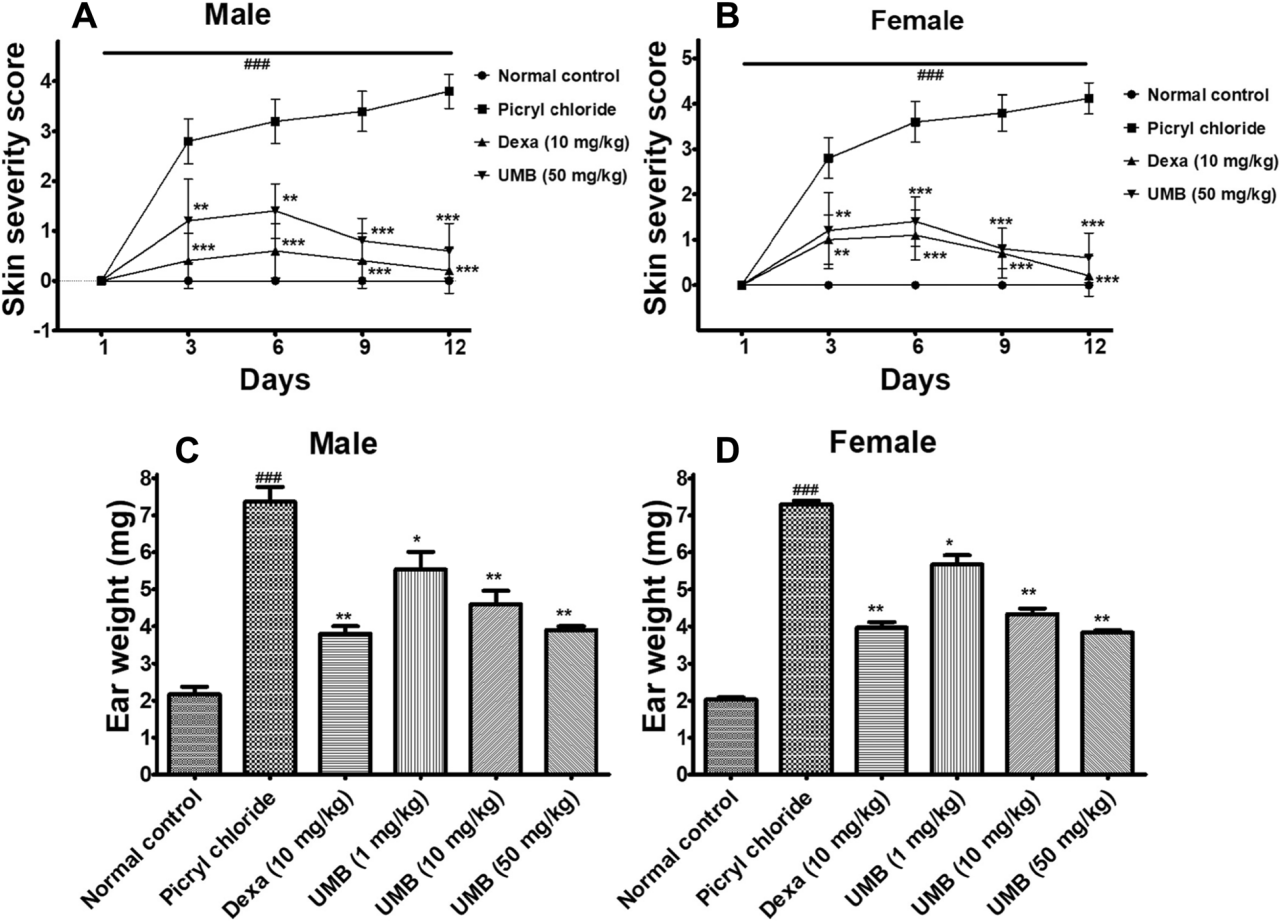
The effect of UMB treatment on skin severity score (**A**, **B**) and ear weight (**C**, **D**) following Picryl chloride (sensitized with Toluene)-induced allergy in both male and female mice. The UMB administration dose dependently reduced the skin severity and ear weight compared to the negative control. The data were represented as mean ± standard deviation (*n* = 5). (*) *p*<0.05, (**) *p*<0.01, (***) *p*<0.001 indicates significance, while ### shows comparison with the negative control

Topical application of PiCl (sensitized with Toluene) showed an increase in skin severity of the negative control group (in both male and female mice). However, a more significant (*P* < 0.001) reduction in the skin severity score was observed in the UMB (50 mg/kg) treated group as compared to the negative control. Furthermore, dexamethasone (10 mg/kg) treated group also showed significant reduction (*P* < 0.001) in the ear edema as shown in Fig. 3.

### Ear weight

The negative control (PiCl sensitized with the Toluene) showed significant increase in the ear weight, however, the UMB treated group showed significant (*P* < 0.05) decrease in the ear weight compared to the negative control (in both male and female mice) as shown in Fig. 3.

### Mice weight

**Fig. 4:**
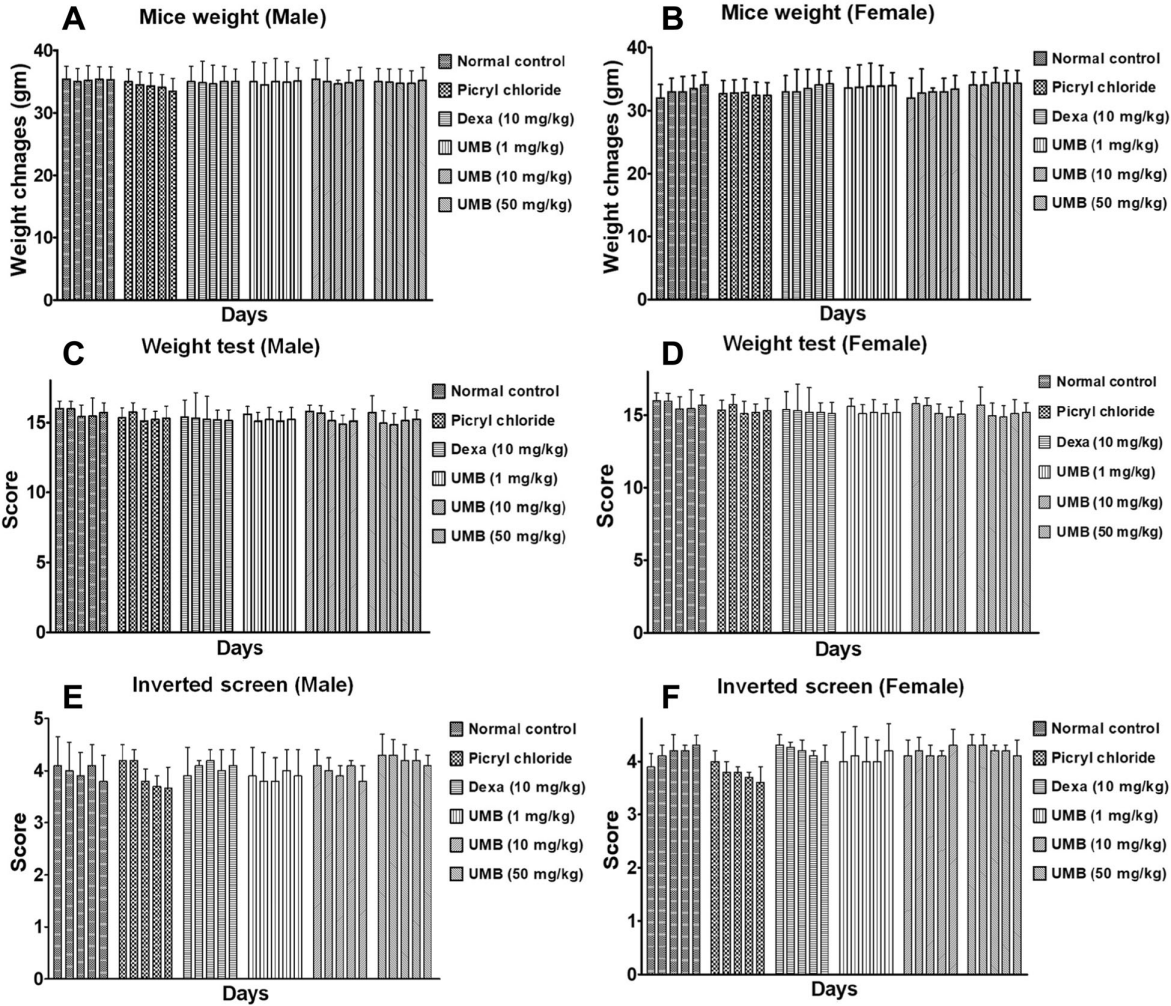
Effect of UMB on mice weight (**A**, **B**), weight lifting (**C**, **D**) and inverted mesh screen (**E**, **F**) following PiCl (sensitized with Toluene) induced ear edema in both male and female mice. No changes were observed in the mice weight and locomotor activity of all the treated groups. The data were represented as mean ± standard deviation (*n* = 5). (*) *p*<0.05, (**) *p*<0.01, (***) *p*<0.001 indicates significance, while ### shows comparison with the negative control

The weight changes in all the treated groups were assessed in case of chronic study (PiCl sensitized with Toluene) and the effect of the UMB treatment was evaluated (in both male and female mice). All the recruited groups treated group showed no changes in the body weights as shown in the Fig. 4.

### Inverted mesh screen and weight lifting assay

The muscle strength and coordination assay was performed to investigate the effect of the UMB treatment on muscle strength and coordination following allergy induction with the PiCl [41]. The muscle strength was determined by the ability of the mice to hold the chain for 3 s [41]. Similarly, the inverted mesh screen test was also used to assess the muscle strength and coordination. However, the result showed no significant changes in the muscle strength and coordination as shown in the Fig. 4.

### Effect of UMB on biochemical and hematological parameters

**Fig. 5:**
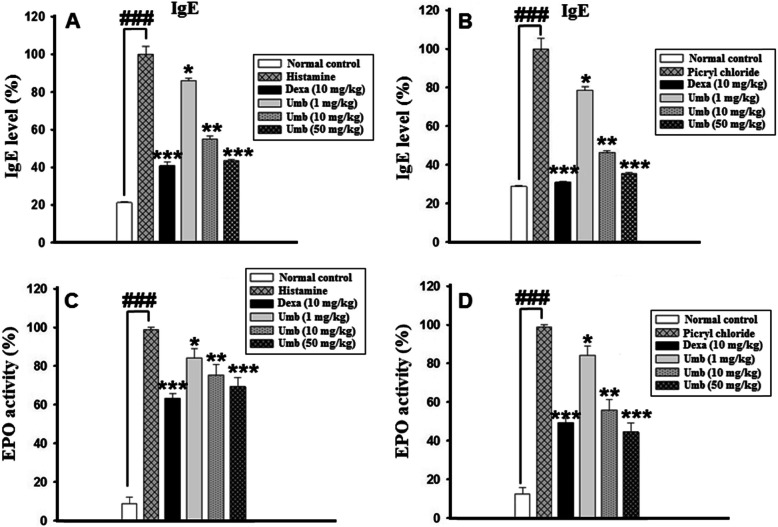
The effect of UMB on serum IgE level in **(A)** histamine and **(B)** PiCl + Toluene-induced allergic ear edema. The UMB treatment dose dependently attenuated the IgE serum level. Furthermore, the effect of the UMB treatment on the EPO activity in both histamine **(C)** and Picryl chloride-induced **(D)** allergic ear edema. The data were represented as mean ± standard deviation (*n* = 5). (*) *p*<0.05, (**) *p*<0.01, (***) *p*<0.001 indicates significance, while ### shows comparison with the negative control

The UMB treatment showed marked (*P* < 0.05) improvement in the hematological parameters compared to the negative control as shown in the Table 1. Similarly, the biochemical parameters such as liver function tests (LFTs) and renal function tests (RFTs) were significantly alter by the UMB treatment as shown in the Table 2. The UMB treatment significantly attenuated the serum IgE level using ELISA kit compared to the negative control as shown in the Fig. 5.

**Table 1:** Hematological parameters of allergic ear edema induced by PiCl+Toluene topical application

Hematological Parameters	Normal control (mean ± SEM)	Picryl chloride (mean ± SEM)	Dexa (10 mg/kg) (mean ± SEM)	UMB (1 mg/kg) (mean ± SEM)	UMB (10 mg/kg) (mean ± SEM)	UMB (50 mg/kg) (mean ± SEM)
Eosinophil’s (%)	0.62 ± 0.23	6.7 ± 0.08^**###**^	4.3 ± 0.16	5.70 ± 0.34	5.1 ± 0.38^**^	4.7 ± 0.32^***^
Basophils (%)	0.3 ± 0.44	0.6 ± 0.034^**###**^	0.32 ± 0.13	0.5 ± 0.42	0.43 ± 0.51^**^	0.33 ± 0.40^***^
Neutrophils (%)	23.5 ± 1.53	53 ± 0.13^**###**^	31 ± 0.36	39 ± 0.28	37 ± 0.45^**^	34 ± 0.45^***^
Monocytes (%)	3.5 ± 0.07	5.3 ± 0.11^**###**^	4.9 ± 0.43	5.1 ± 0.47	4.7 ± 0.21^**^	4.8 ± 0.20^***^
WBC (×10^3^/mm^3^)	2.7 ± 0.29	3.5 ± 0.50^**###**^	2.7 ± 021	2.6 ± 0.61	2.8 ± 0.41^**^	2.9 ± 0.65^***^

The data were represented as mean ± standard deviation (*n* = 5). (*) *p*<0.05, (**) *p*<0.01, (***) *p*<0.001 indicates significance, while ### shows comparison with the negative control

**Table 2:** Various Biochemical Parameters were observed in PiCl+Toluene induced chronic allergic model

Biochemical Parameters	Normal (mean ± SEM)	Picryl chloride (mean ± SEM)	Dexa (10 mg/kg) (mean ± SEM)	UMB (1 mg/kg) (mean ± SEM)	UMB (10 mg/kg) (mean ± SEM)	UMB (50 mg/kg) (mean ± SEM)
ALT (U/L)	26.50 ± 5.76	28 ± 6.99^**###**^	29 ± 7.19	28 ± 8.15	27 ± 4.33	29 ± 3.21
AST (U/L)	70.49 ± 8.46	80.7 ± 9.65^**###**^	75.21 ± 13.4	74.38 ± 6.99	75.0 ± 7.38	74.61 ± 8.43
Bilirubin (mg/dL)	2.41 ± 0.25	2.67 ± 0.15^**###**^	2.39 ± 0.20	2.32 ± 0.11	2.41 ± 0.03	2.37 ± 0.02
Creatinine (U/L)	0.22 ± 0.21	0.25 ± 0.34^**###**^	0.23 ± 0.03	0.24 ± 0.04	0.25 ± 0.45	0.26 ± 0.52

The data were represented as mean ± standard deviation (*n* = 5). (*) *p*<0.05, (**) *p*<0.01, (***) *p*<0.001 indicates significance, while ### shows comparison with the negative control

### Effect of UMB on antioxidants and oxidative stress markers

The negative control groups showed a sharp reduction in the antioxidants enzymes such as GST, GSH, Catalase and SOD in the ear homogenate, however, the UMB treatment significantly enhanced the antioxidants enzymes as shown in the Fig. 7. Furthermore, the effect of the UMB treatment was also studied on the oxidative stress markers such as MDA following allergy induction with the PiCl. The UMB treatment significantly attenuated the production of the MDA i.e. the product of the lipid peroxidation compared to the negative control Fig. 8.

**Fig. 6:**
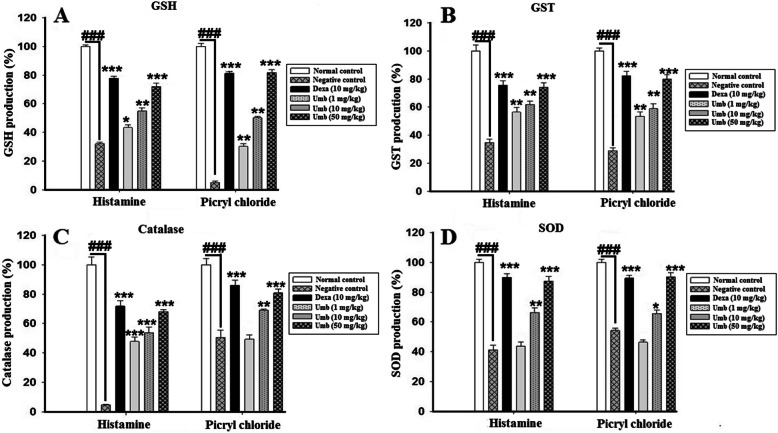
The effect of the UMB treatment on the acute and chronic ear edema-induced changes in the antioxidants enzymes. The UMB treatment markedly induced the antioxidants enzymes such as GSH **(A)**, GST **(B)**, Catalase **(C)**, and SOD **(D)** in the ear tissue in both acute and chronic allergy models. The data were represented as mean ± standard deviation (*n* = 5). (*) *p*<0.05, (**) *p*<0.01, (***) *p*<0.001 indicates significance, while ### shows comparison with the negative control The data were represented as mean ± standard deviation (*n* = 5). (*) *p*<0.05, (**) *p*<0.01, (***) *p*<0.001 indicates significance, while ### shows comparison with the negative control

**Fig. 7:**
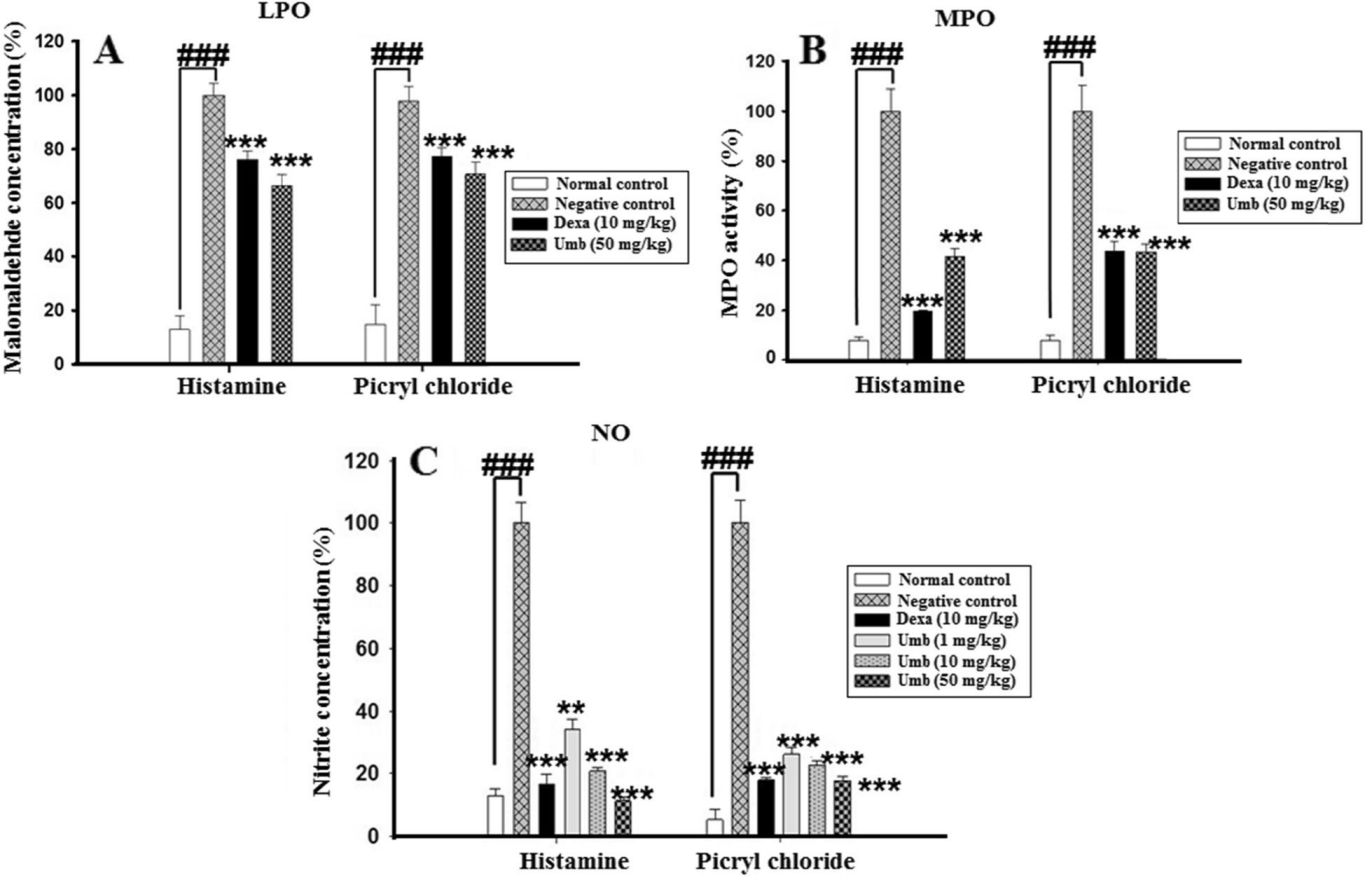
The influence of UMB treatment on the MDA **(A)**, MPO **(B)**, and NO **(C)** production in the ear homogenate in both acute and chronic ear allergy model. The UMB treatment markedly attenuated the MDA, MPO and NO production compared to the negative control. The data were represented as mean ± standard deviation (*n* = 5). (*) *p*<0.05, (**) *p*<0.01, (***) *p*<0.001 indicates significance, while ### shows comparison with the negative control

### Effect of UMB on NO production

**Fig. 8:**
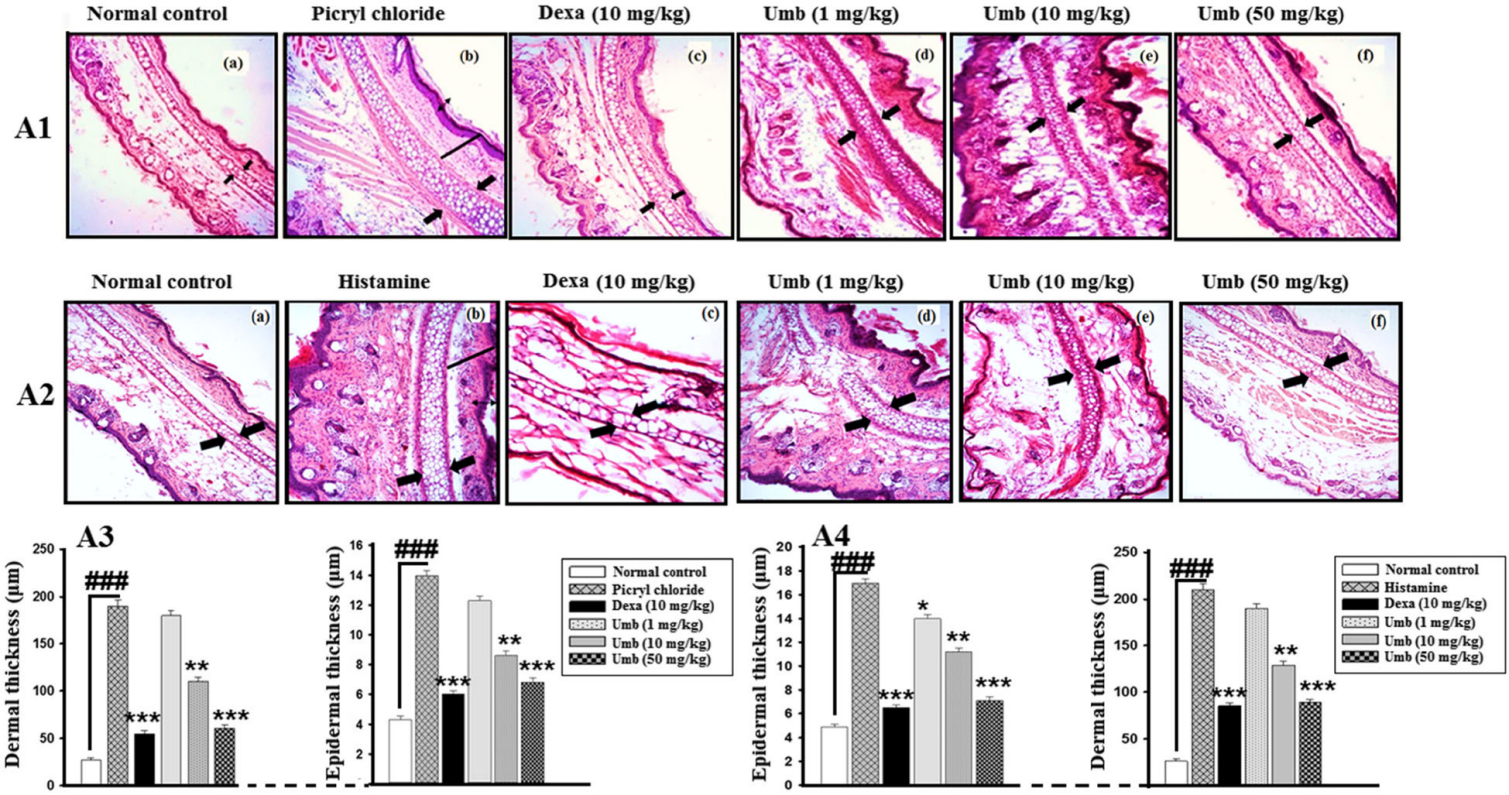
The influence of UMB treatment on the histological parameters in both histamine (**A**) and Picryl chloride-induced (**B**) ear allergy. The UMB (1, 10, 50 mg/kg) treatment showed significant improvement in the histopathology in both acute and chronic models. Similarly, the dexamethasone also improved the histological parameters compared to the negative control. The data were represented as mean ± standard deviation (*n* = 5). (*) *p*<0.05, (**) *p*<0.01, (***) *p*<0.001 indicates significance, while ### shows comparison with the negative control

The concentration of NO was found higher in tissue homogenate of negative control group following induction of ear edema, however, the UMB treatment markedly attenuated the NO production compared to the negative control. Similarly, the positive control treated with the dexamethasone also markedly attenuated the NO production in both plasma and ear homogenate compared to the negative control as shown in Fig. 8.

### Effect of UMB on MPO activity

The MPO activity (indirect marker of neutrophilic infiltration) was significantly raised in the ear homogenate of the negative control group. However, the UMB (50 mg/kg, i.p) treated group showed a marked reduction in the MPO activity in the ear homogenate in contrast to the negative control. Similarly, the dexamethasone also remarkably attenuated the MPO activity compared to the negative control as shown in Fig. 8.

### Effect of UMB treatment on EPO activity

Similarly, the EPO assay (an indirect indicator of the eosinophil infiltration) was performed to assess the effect of UMB treatment on the EPO activity following ear edema induction. The negative control showed marked increase in the EPO activity, however, the UMB treatment remarkably inhibited the activity of the EPO compared to the negative control as shown in Fig. 5.

### Effect of UMB treatment on histological parameters

**Fig. 9:**
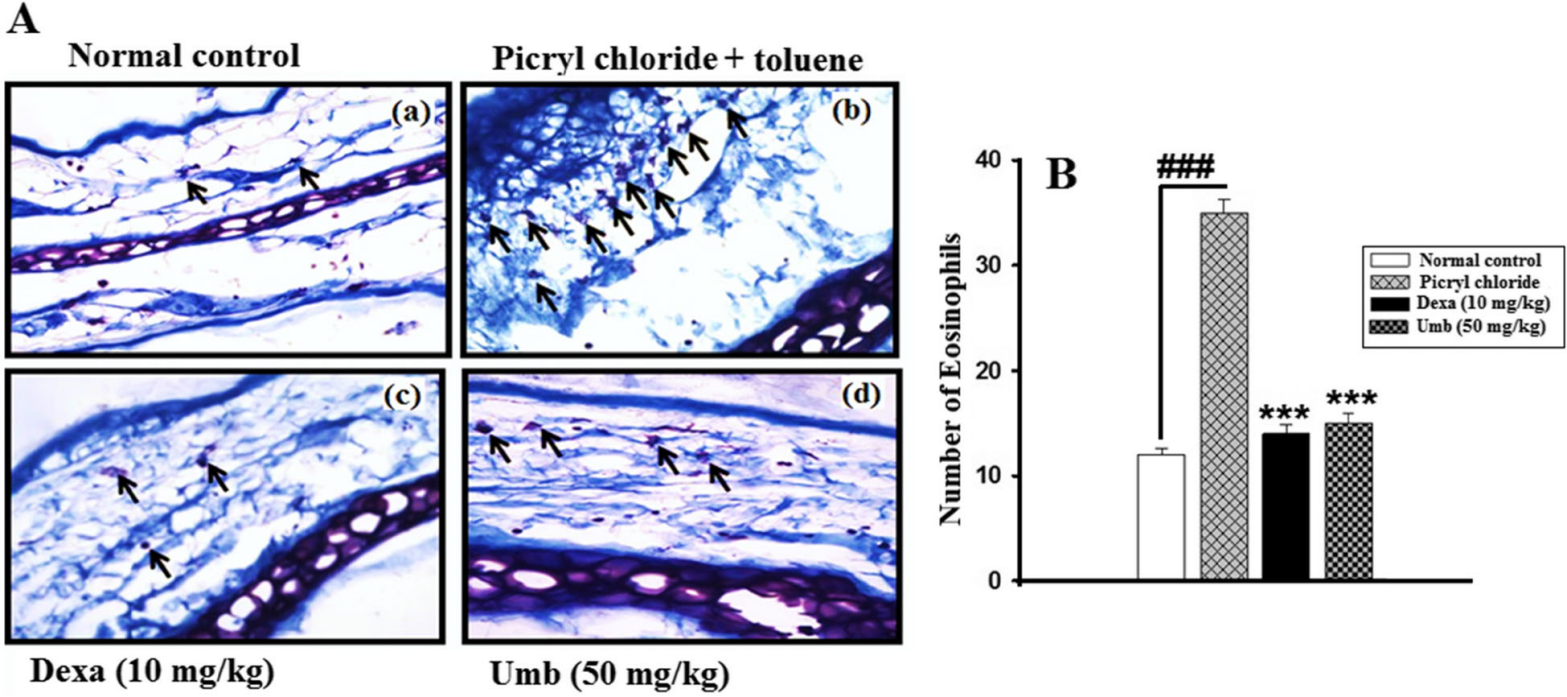
Giemsa staining for the assessment of eosinophilic infiltration in the chronic PiCl (sensitized with the Toluene)-induced allergy and the subsequently the evaluation of the UMB treatment on the allergic ear edema. The UMB treatment showed significant improvement in the Giemsa staining compared to the negative control. The data were represented as mean ± standard deviation (*n* = 5). (*) *p*<0.05, (**) *p*<0.01, (***) *p*<0.001 indicates significance, while ### shows the comparison with the negative control

**Fig. 10:**
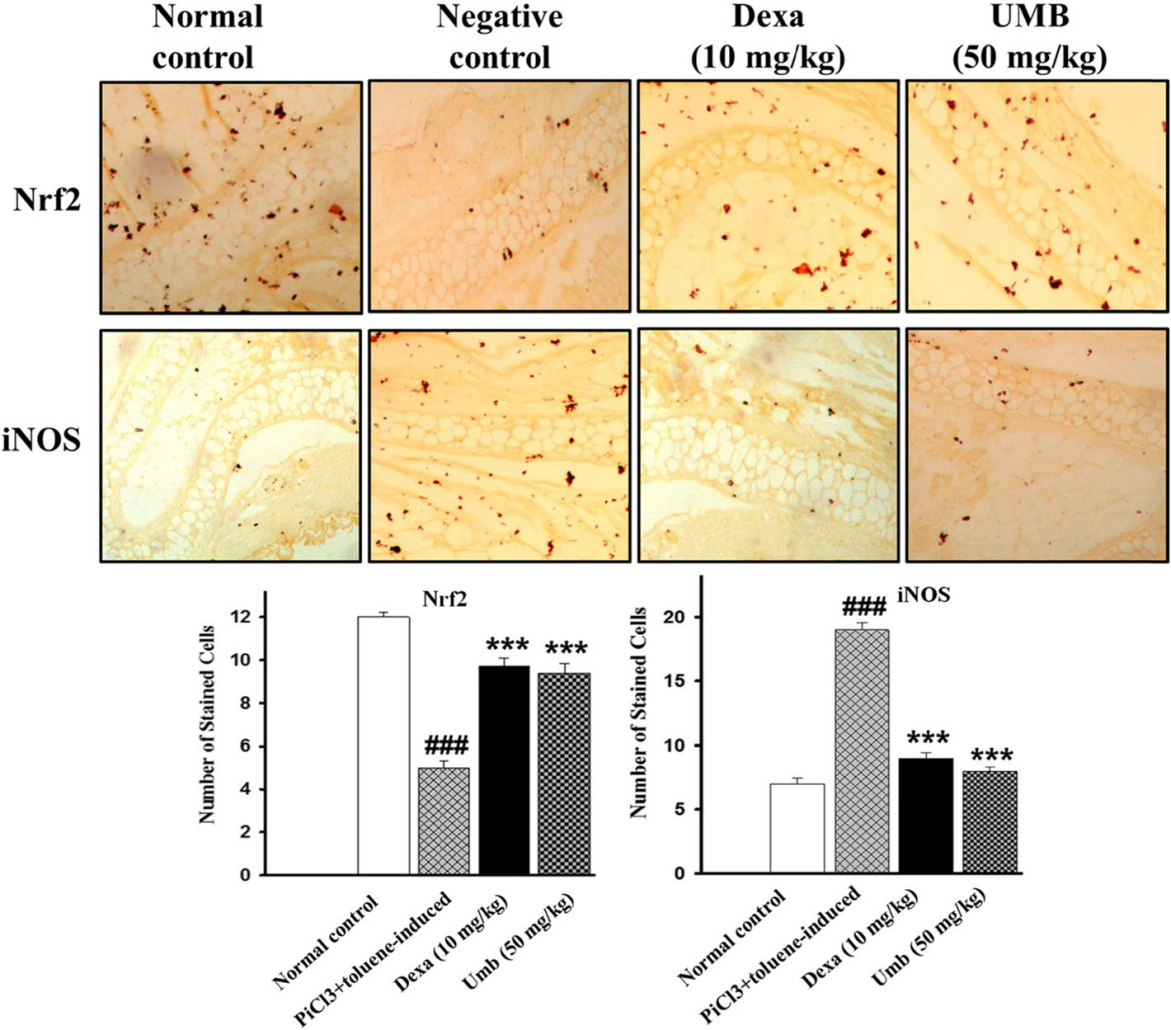
Effect of UMB treatment on the Nrf2 and iNOS expression level in ear tissue following Picryl chloride-induced ear edema. The UMB treatment markedly attenuated the iNOS expression level, while induced the Nrf2 protein expression level compared to negative control. The data were represented as mean ± standard deviation (*n* = 5). (*) *p*<0.05, (**) *p*<0.01, (***) *p*<0.001 indicates significance, while ### shows comparison with the negative control

The H & E staining showed epidermal and dermal hyperplasia along with increase in inflammatory cells in the negative control group Fig. 9. The UMB (50 mg/kg) treated group showed significant improvement in the histopathology compared to the negative control. Furthermore, the Giemsa staining was also performed to assess the eosinophilic infiltration in all the treated groups. The Giemsa staining showed a remarkable increase in the eosinophilic infiltration in the negative control group. However, the UMB treated group marginally reversed the eosinophils compared to the negative control as shown in Fig. 10.

### Effect of UMB on Immunohistopathological staining

Immunohistochemistry analysis revealed marked increase in the expression of iNOS protein, while attenuated the Nrf2 expression level in the PiCl treated group (sensitized with the Toluene). However, the UMB treatment enhanced the expression level of Nrf2 and attenuated the iNOS expression level as shown in the Fig. 6.

### Bioinformatics analysis

**Fig. 11:**
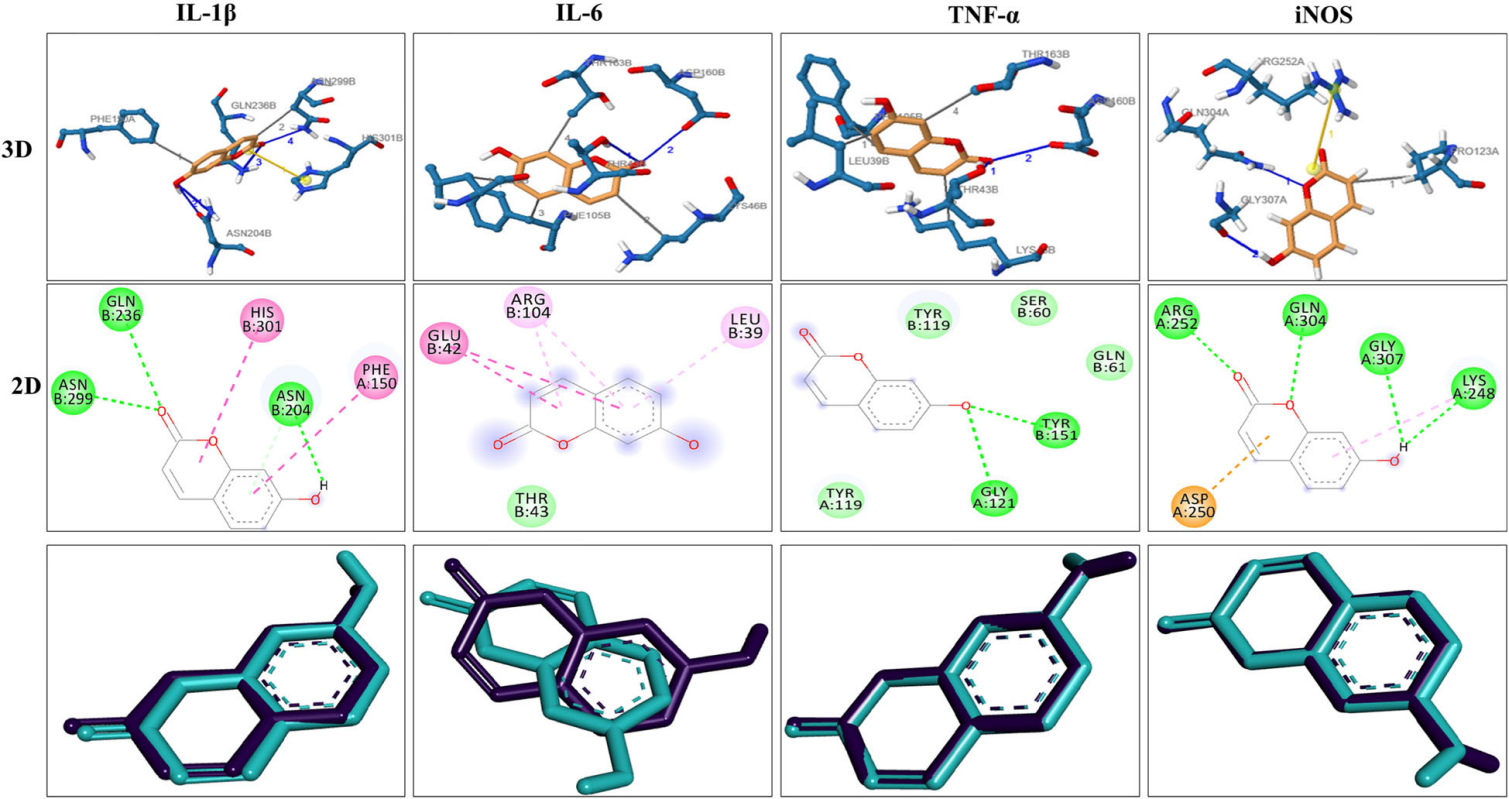
Determination of compatibility of UMB at various target sites (IL-1β, IL-6 and TNF-α) involved in the process of allergic inflammation through Auto-Dock and MOE. The yellow colour shows UMB (ligand), while the light magenta colour shows the active site residues. The interacting amino acids are shown as green sticks. The 3D, 2D and docking validation structures are exhibited respectively

**Fig. 12:**
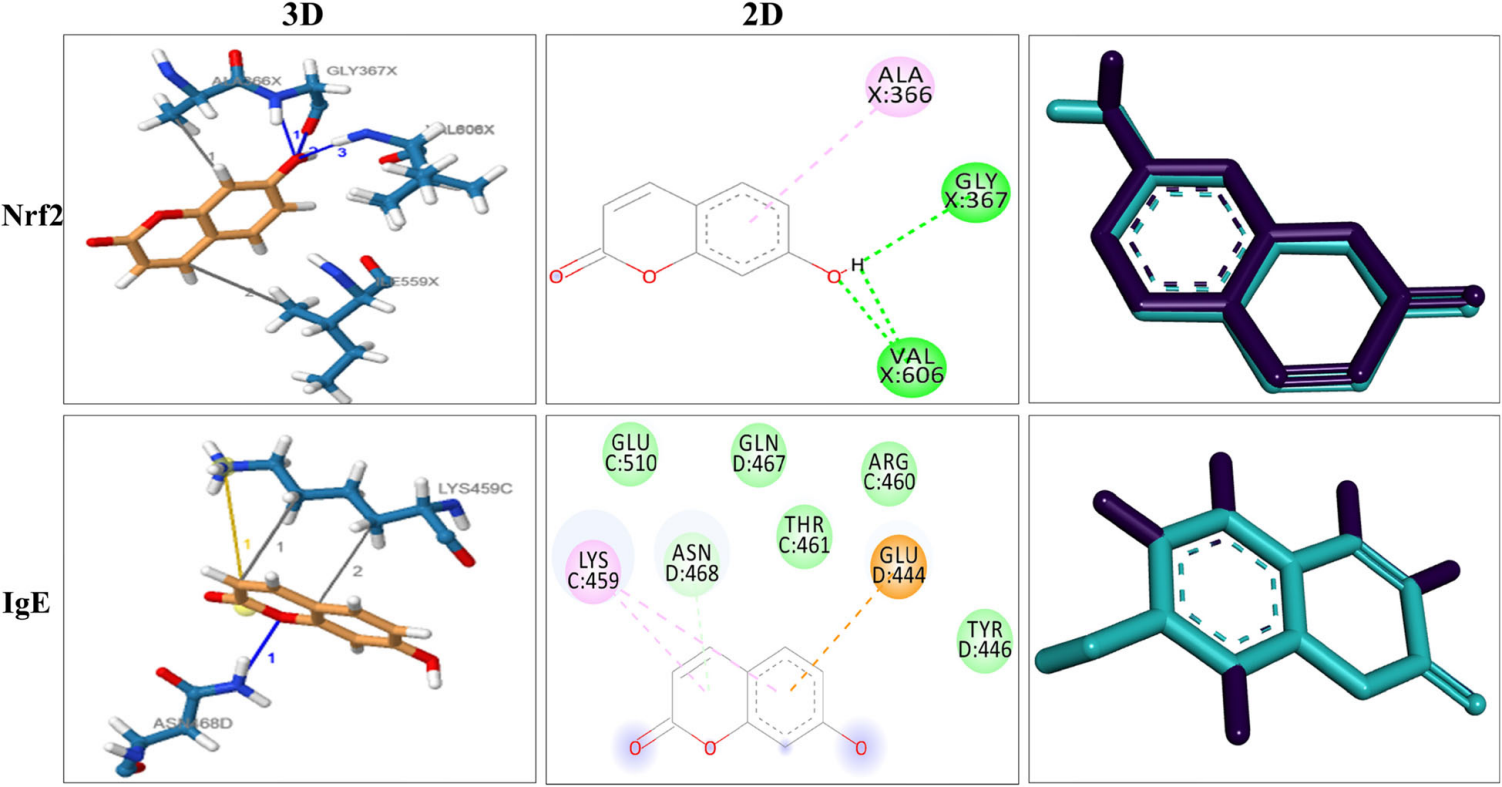
Molecular docking analysis of UMB (ligand) with the Nrf2 and IgE protein. The docking analysis showed the interaction of the UMB with various proteins via hydrogen bonds and hydrophobic bonds. The 3D and 2D interaction of the ligand with the protein targets and validation of the docking are represented

The UMB showed marked interaction with the IL-1β, IL-6, TNF-α, IgE, Nrf2 and iNOS proteins via multiple hydrogen and hydrophobic bonds. The 3D and 2D structure of UMB interaction with their protein targets Figs. 11 and 12, while the number hydrogen bonds, interacting amino acids, molecular docking energy and RMSD are shown in the Table 3.

**Table 3:** The docking interaction of the UMB with various protein targets

Proteins	PDB ID	RMSD (Å)	H-bonds amino acids	H-bonds numbers	Binding energies (kcal\mol)
**TNF-α**	2az5	0.319	Gly121, Tyr151	2	−6.9
**IL-1β**	1itb	0.289	Asn204, Gln236, Asn299	3	−7.3
**IL-6**	1p9m	1.21			−6.3
**iNOS**	1dd7	0.308	Lys248, Gly307, Gln304, Arg252	4	−6.8
**Nrf2**	2flu	0.230	Gly367, Val606, Ala666	3	−6.7
**IgE**	4grg	0.254	Asn468	1	−6.8

### Pharmacokinetics analysis

**Fig. 13:**
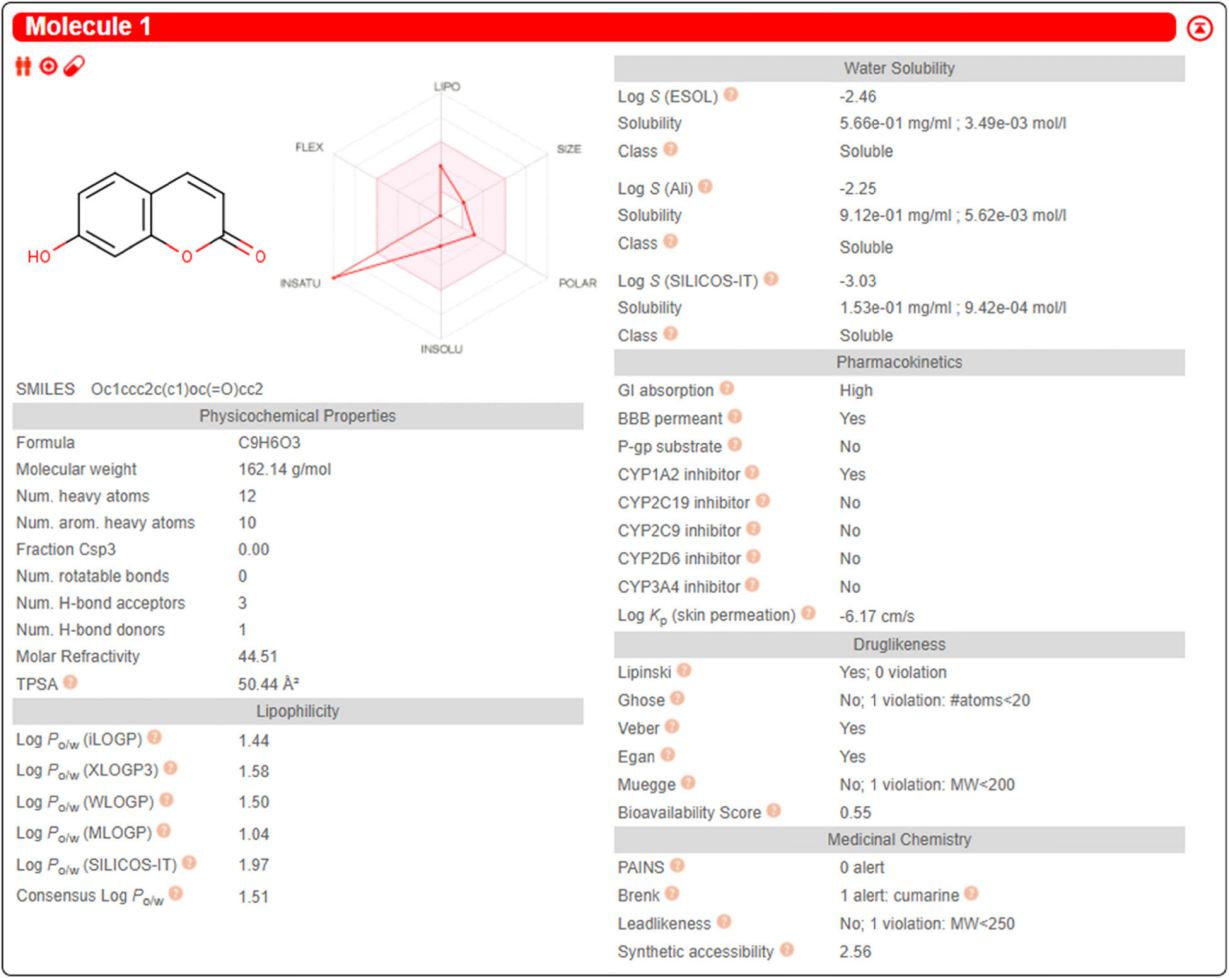
The pharmacokinetic assessment of the UMB using in silico approaches. The Swiss target prediction online software was used to assess the pharmacokinetic parameters of the UMB. The various properties that were taken into consideration include physicochemical properties, liophilicity, water-solubility, pharmacokinetics, drug-likeness and medicinal chemistry aspects. The UMB overall showed better pharmacokinetic and physicochemical behavior

The UMB physicochemical and pharmacokinetic analysis was determined using in silico approaches [39, 40]. The UMB showed promising physicochemical properties such as logP, water-solubility, lipid solubility, and drug likeness behavior. Similarly, the UMB showed encouraging bioavailability, absorption, distribution, showed metabolism via cytochrome p450 system and excretion via renal route as shown in the Fig. 13. Furthermore, the UMB portrayed promising drug likeness and medicinal chemistry properties as shown in the Fig. 13.

## Discussion

Umbelliferone (UMB) is a coumarin derivative found in various parts of the plants such as flowers, roots and stem isolated from the plants of Umbelliferae family. The UMB has been reported for various biological properties such as anti-inflammatory, anti-diabetic and anti-fungal, however, the anti-allergic activities has not been reported yet [30]. In the current study, the UMB was investigated against both acute (histamine) and chronic (PiCl+Toluene) ear edema models in mice. The histamine is well known mediator of allergic inflammation and released when the body is hypersensitive to any substance [31, 42]. The acute ear edema was induced by administering the histamine (10 mg/kg, i.d), while the chronic ear edema was induced by multiple topical application of the PiCl (sensitized with the Toluene) at the dorsal surface of ear [21, 31, 43]. During acute histamine-induced ear edema the UMB (UMB administration 30 min after the histamine-induced ear edema) dose dependently reduced the ear edema compared to the negative control. Similarly, the negative control showed marked increase in the ear edema following PiCl (sensitized with the Toluene), however, the UMB treatment dose dependently reduced the ear edema and the dose of 50 mg/kg showed maximum response. Similarly, the dexamethasone also attenuated the allergic ear edema following induction of ear edema with the PiCl+Toluene compared to the negative control. The UMB treated group showed remarkable improvement in the behavioral parameters such as allergic clinical scoring compared to the negative control. The histamine and PiCl administration significantly altered the antioxidants enzymes (GST, GSH, Catalase and SOD) and oxidative stress markers i.e. MDA. While treatment with UMB markedly induced the antioxidants enzymes and neutralized the oxidative stress markers in ear homogenate compared to the negative control. The NO an important mediator of inflammation and influenced by the iNOS enzyme activity [44, 45]. During inflammatory and allergic conditions the NO concentration raised proportionally [46, 47]. In the current study, the NO production was significantly elevated in the negative control group (PiCl+Toluene induced allergy), however, the UMB treatment showed significant decrease in the NO production compared to the negative control. Similarly, the MPO activity (marker of neutrophil infiltration) raised significantly during the inflammation and allergy [48, 49]. The MPO assay was performed to assess the effect of the UMB treatment on the neutrophilic infiltration following chronic ear edema induction with the PiCl+Toluene [46, 48]. The UMB treatment markedly attenuated the MPO activity compared to the negative control. Furthermore, the EPO activity indirectly indicates the eosinophils infiltration to site if injury and commonly implicated in the allergic diseases [48, 50]. In the present study, the PiCl administration markedly raised the EPO concentration, however, UMB treatment significantly attenuated the EPO concentration compared to the negative control.

The changes in the hematological parameters are commonly implicated during inflammatory and allergic conditions [48, 50]. The blood complete analysis was performed to assess the effect of the UMB treatment on blood composition following ear edema induction with the PiCl+Toluene. The negative control showed marked elevation in both neutrophils and eosinophils count, however, the UMB treatment significantly reduced the neutrophilic and eosinophilic count in the blood and improved other hematological parameters compared to the negative control. Similarly, serum analysis was performed to investigate the changes in liver and kidney functions test following allergic ear edema induced with the PiCl+Toluene [51, 52]. The LFTs and RFTs data showed no observable changes in all the treated groups included UMB.

In order to evaluate the histological changes in the ear histology following induction of acute and chronic allergy and subsequently to assess the effect of the UMB treatment on the histological parameters H and E staining was performed [53]. The H and E staining showed that UMB treatment markedly reduced the epidermal edema, hypertrophy and immune cells infiltration compared to the negative control (both acute histamine and chronic PiCl+Toluene-induced allergy). Similarly, the effect of the UMB treatment on the eosinophilic infiltration to the site of allergic inflammation was studied using Giemsa staining [53, 54]. The Giemsa staining showed a significant decrease in the eosinophilic infiltration in the UMB treated group compared to the negative control. Furthermore, the positive control group treated with the dexamethasone also improved the histological parameters compared to the negative control.

Serum IgE antibodies are key marker of Type 1 hypersensitivity reaction in allergic diseases including atopic dermatitis [51]. Allergen specific IgE binds to its receptor (FcεRI and FcεRII or CD23) on mast and basophil cells [54]. The disparity of T helper 1 (Th1) and T helper 2 (Th2) cells have been reported in atopic dermatitis with the dominancy of the T helper 2 responses, which leads to the increase production of IgE antibodies [54]. For this purpose, levels of IgE were analyzed in both acute and chronic allergy models. The UMB treatment markedly reduced the serum IgE level compared to the negative control. The computational tools are attracting the attention of the researchers and serves to assess the mechanism of interaction of the ligand with protein targets. The computational analysis solved many mysteries associated with the interaction of ligand and proteins [47, 55]. In the current study, the computational analysis was used to assess the mechanism and validate the interaction of the ligand with the protein targets. The UMB interacted with the protein targets via multiple hydrogen bonds and hydrophobic bonds. Similarly, the molecular docking interaction of the ligand with the protein targets was also validated. The immunohistochemistry was performed to assess the effect of the UMB treatment on the expression level of iNOS and Nrf2 protein following allergy induction with the PiCl+Toluene [52, 55]. The negative control group showed marked decrease in the expression of Nrf2, while showed significant increase in the iNOS proteins. However, the UMB treatment markedly attenuated the iNOS protein expression and induced Nrf2 expression compared to the negative control.

## Conclusion

The current study evaluated the anti-allergic activity of the UMB against both acute histamine- and chronic PiCl (sensitized with Toluene)-induced ear edema. The UMB treatment markedly attenuated the ear edema and ear weight compared to the negative control. The UMB treatment induced the antioxidants enzymes and attenuated the oxidative stress markers. The UMB treatment significantly improved the histological parameters and reduced the immune cells infiltration to the site of allergic inflammation. The UMB reduced the iNOS expression, while induced the expression of Nrf2 proteins. In short, the UMB treatment significantly reduced the allergic ear edema and improved the sign and symptoms associated with the histamine and PiCl (sensitized with Toluene)-induced ear edema.

## Data Availability

The data presented in this study are confined and described within the article and will be disseminated by the corresponding author upon request. All materials used in this study were included in methods section adequately.

## Abbreviations

UMB: Umbelliferone
PiCl: Picryl chloride
Nrf2: Nuclear factor erythroid 2-related factor 2
TNF-α: Tumor necrosis factor-α
GST: Glutathione S-transferases
MPO: Myeloperoxidase
EPO: Eosinophil peroxidase
NO: Nitric oxide
SOD: Sulphur oxide dismutase
LPO: Lipid peroxidase
HO-1: Hemeoxygenase-1
MDA: Malonaldehde
IL-6: Interleukin 6
IL-1β: Interleukin-1β
iNOS: Inducible nitric oxide synthase
